# Social Touch Gesture Recognition Using Convolutional Neural Network

**DOI:** 10.1155/2018/6973103

**Published:** 2018-10-08

**Authors:** Saad Albawi, Oguz Bayat, Saad Al-Azawi, Osman N. Ucan

**Affiliations:** ^1^Altinbas University, Graduate School of Science and Engineering, Istanbul, Turkey; ^2^University of Diyala, College of Engineering, Diyala, Iraq

## Abstract

Recently, social touch gesture recognition has been considered an important topic for touch modality, which can lead to highly efficient and realistic human-robot interaction. In this paper, a deep convolutional neural network is selected to implement a social touch recognition system for raw input samples (sensor data) only. The touch gesture recognition is performed using a dataset previously measured with numerous subjects that perform varying social gestures. This dataset is dubbed as the corpus of social touch, where touch was performed on a mannequin arm. A leave-one-subject-out cross-validation method is used to evaluate system performance. The proposed method can recognize gestures in nearly real time after acquiring a minimum number of frames (the average range of frame length was from 0.2% to 4.19% from the original frame lengths) with a classification accuracy of 63.7%. The achieved classification accuracy is competitive in terms of the performance of existing algorithms. Furthermore, the proposed system outperforms other classification algorithms in terms of classification ratio and touch recognition time without data preprocessing for the same dataset.

## 1. Introduction

Social touch is one of the basic interpersonal methods used to communicate emotions. Social touch classification is a leading research area which has great potential for further improvement and development [1]. Social touch classification can benefit human-robot interaction [2]. The identification of the type (or class) of touch when a human touches a robot's artificial skin is a demanding yet simple question in this area [3, 4]. A human can easily distinguish and understand social touch. However, an interface with which to record social touch should be developed in human-robot interaction [5–7]. Several attempts have been made to build devices to classify human social touch and record them for the available dataset [8–15]. This paper concentrates on the existing studies that proposed a setup with which to measure the pressure of touch of recorded data and recognize the classes of social touch gesture. This setup, which is used to record the corpus of social touch (CoST), is a type of artificial skin that records the pressure applied on it.

Previous studies have aimed to identify the touch classes using 14 predefined classes [5–10, 12, 16]. These social gestures consist of grab, hit, massage, pat, pinch, poke, press, rub, scratch, slap, stroke, squeeze, tap, and tickle, which were taken from the Yohanan dictionary [16]. However, they do not satisfy a fully real-time system. Note that even humans need to wait a certain amount of time (e.g., in the order of milliseconds) to understand social touch class [2]. Therefore, this paper aims to classify social touch in a reasonably short time. Consequently, the amount of data (number of frames) on average is necessary to recognize social gestures.

Another issue is the avoidance of preprocessing, which develops case dependency, and, as previously discussed, prevents real-time performance (e.g., using an average or any measurement which performs temporal abstraction) [17]. This paper introduces a model for the social touch recognition which avoids the data preprocessing step. Thus, the study explores the question “How can social touch be classified by providing raw input samples (sensor data) only instead of a set of features?” Furthermore, the use of sensor data without preprocessing is a challenged task, such that a powerful approach to efficiently classify gesture classes is required.

To handle this huge amount of data, we use a robust tool, which has become popular in the literature [18–20]. Convolutional neural networks (CNN) constitute multiple layers of artificial neural networks that currently surpass classical methods in performance, such as pattern recognition and image and object detection, in other fields [21–23]. The key points of the proposed method are as follows:

- High performance and accuracy, which outperforms other recognition algorithms applied to the same dataset
- A CNN is used to recognize social gestures in an end-to-end architecture
- No preprocessing operations are required (except rescaling pressure data between 0 and 1 by dividing them to 1,023, which is the maximum measureable pressure)
- Classification operation starts after receiving a minimum number of frames (frame length = 85)
- Social gesture class is predicted in nearly real time, after 629 ms of the raw input samples (sensor data)
- Gestures are classified even if the data sample is given in the middle of the gesture

The remainder of this paper is organized as follows: Section 2 gives a brief introduction to the CoST dataset and convolutional neural network.  Section 3 describes the architecture of our proposed convolutional neural network.  Section 4 presents the results and discussion of the proposed method.  Section 5 summarizes the main findings and proposes future research.

## 2. Background

This section introduces the CoST, describes the CNN, and sets up the parameters used to build the network.

### 2.1. CoST Dataset

**Figure 1:**
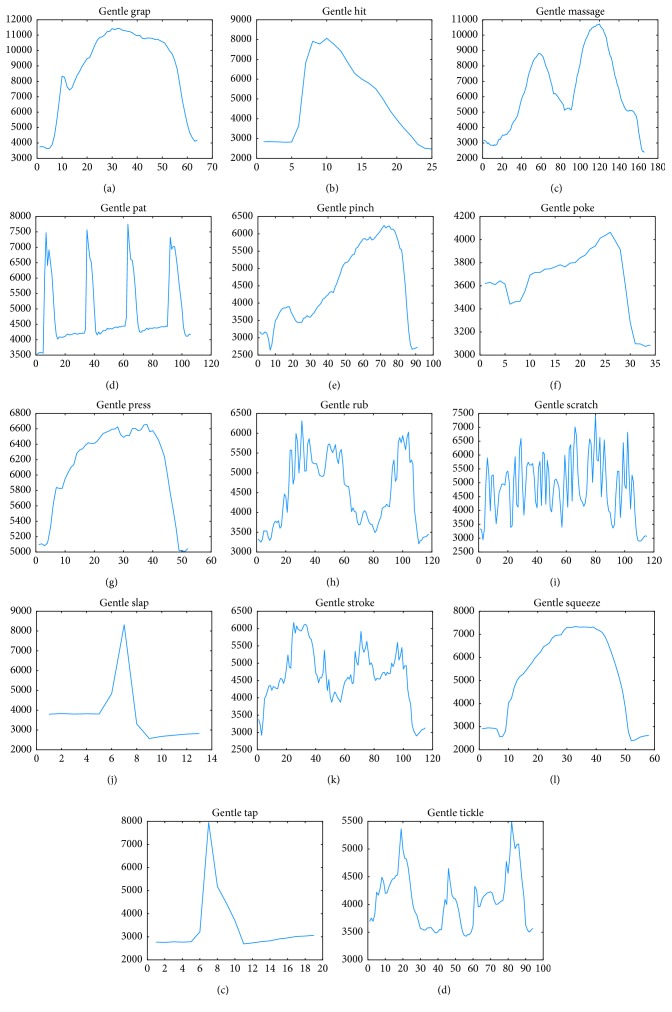
Gesture instances for each class for time (*x*-axis) and summed pressure (*y*-axis).

The CoST dataset provides recorded social touch gestures from various subjects. The data frame was collected using a pressure sensor installed in the mannequin arm. The pressure sensor grid detectable pressure ranges from 1.8 × 10^−3^ to >0.1 megaPascal (MPa) at an ambient temperature of 25°C. In an 8 × 8 grid, which covers the artificial skin, the sensor data were sampled at 135 Hz (frame per second). A single experiment collected from a subject consists of an 8 × 8 × *N* matrix (where *N* is the number of frames or frame length, as called in this paper). The experiment was conducted on 31 subjects (24 males and 7 females). A total of 14 social gestures, namely, grab, hit, massage, pat, pinch, poke, press, rub, scratch, slap, stroke, squeeze, tap, and, tickle [16], were recorded. The subjects were also asked to perform each gesture in gentle, normal, and rough variations, with each variation repeated six times. In total, each subject performed 252 gestures, and the CoST collected 7,805 gestures from the subjects (few gestures are missing from the dataset) [8, 9].  Figure 1 shows the summed pressure (*y*-axis) with respect to a period of time (*x*-axis) for gestures for one subject (#1) who performs 14 gentle variation gestures (data available on request, https://doi.org/10.4121/uuid:5ef62345-3b3e-479c-8e1d-c922748c9b29).

Several studies have classified the CoST dataset using various classification methods depending on distinct number of features extracted from raw input data. The first method was introduced by Jung et al. [8, 9] using the Bayesian classifier and support vector machine (SVM) for classification.

They used 28 features extracted from the dataset based on mean and maximum pressure, pressure variability, mean pressure per column and row, contact area, peak count, displacement, and duration. Classification results were evaluated using leave-one-subject-out cross validation. Results of touch gesture recognition ranged from 24% to 75% (*M* = 54%, SD = 12%) for Bayesian classifiers and from 32% to 75% (*M* = 53%, SD = 11%) for SVM.

Gaus et al. [12] elicited various types of features. They classified the CoST dataset using random forest (RF) and boosting algorithms. They extracted five sets of high level features, namely, statistical distribution of pressure surface, binary motion history, motion statistical distribution, spatial multiscale motion history histogram on touch dynamics, and local binary pattern on three orthogonal places on touch dynamics). The models were firstly trained on the training subset and evaluated using a 10-fold cross-validation process. The results obtained were 59.5% for RF and 58.1% for the boosting algorithm.

To achieve high accuracy for gesture recognition, Hughes et al. [13] employed gesture-level features. They selected deep autoencoders as the classification method. To estimate the performance of models, they used 10-fold stratified cross validation. They obtained 56% accuracy for gesture recognition. Seven distinct features were extracted from the dataset based on the maximum value of pressure through touch, area of pressure on the sensor, and number of gestures repeated at each touch.

Ta et al. [15] divided 273 features into 3 categories. Global, which consists of 40 features, represents the overall statistics of the gesture. Channel-based consists of 192 features and describes the spatial relationship among a number of channels. The sequence of average pressure consists of 41 features, which utilized the sequence of average pressure over all channels for each frame. A threefold cross-validation method was used to evaluate recognition performance. The gesture recognition accuracy obtained was 60.51% and 60.81% for SVM and RF, respectively.

Hughes et al. [24] applied three different methods of deep learning for social touch recognition on CoST and HAART dataset. For CNNs and CNN-RNNs, the CoST data were split into windows with a window size of 45 samples (333 ms) and a hop size of 15 samples (111 ms). The HAART data were split into windows with a window size of 27 samples (500 ms) and a hop size of 9 samples (167 ms). In order to efficiently train the CNN-RNN model, they limited the number of windows in a training sample to 36, which resulted in some of the gesture captures being split into two or three training samples. The HAART dataset had consistent gesture durations, and each training sample consisted of a complete capture. For the autoencoder-recurrent neural network, they used 7 distinct features taken from [13]. The classification ratios of convolutional neural network used were 42.34% and 56.10% for CoST and HAART dataset, respectively. The classification ratios for a convolutional-recurrent neural network (CNN-RNN) were 52.86% and 61.35%. While for an autoencoder-recurrent neural network, the classification ratios were 33.52% and 61.35%, respectively. The three used methods of deep learning satisfy a similar level of recognition accuracy and make gestures predicted in a short time at a rate of 6 to 9 Hz.

Zhou and Du [25] compared the performance for various types of the deep-learning algorithms for gesture recognition on the human-animal affective robot touch (HAART) dataset, which consists of 7 different gestures (constant, no touch, pat, rub, scratch, stroke, and tickle). The algorithms consists of neural network structures including 2D CNNs, 3D CNNs, and LSTMs. GM-LSTMs, LRCNs, and 3D CNNs are compared on the social touch gestures recognition task. However, the proposed 3D CNN approach when applied on HAART dataset satisfies a recognition accuracy of 76.1%, significantly outperforming the other proposed algorithms. The number of convolutional layers was set to 4. They found the configuration of convolutional layers to 4 layers, the number of filters at each layer set as 16-32-64-128, respectively. 3D CNNs achieved the best performance, compared with 8-16-32-64 and 32-64-128-256. The kernel sizes in every convolutional layers were all set to be 3 × 3 × 3.

Lastly, Jung et al. [17] applied 4 distinct methods and 54 features, such as mean and maximum pressure, pressure variability, mean pressure per row and column, contact area per frame, which were extracted from the dataset. Data were divided into training and testing sets, and leave-one-subject-out cross validation (31 folds) was utilized to evaluate the accuracy of the algorithms. Four methods from machine learning were applied on the CoST dataset to evaluate its performance and classification ratios. The Bayesian classifier achieved 57% (SD = 11%), decision tree algorithm 48% (SD = 10%), and SVM with RBF kernel 60% (SD = 11%), when they used a feed-forward network and trained the network by using Levenberg–Marguardt optimization method. Stopping criteria were set to a maximum of 1000 training iterations or six subsequent increases of the error on the validation set. Because of memory constraints, the architecture was set to two layers of 54 and 27 neurons. The subjects were split into a train set 70% and a validation set 30%; the accuracy of classification was 59% (SD = 12%).

### 2.2. Convolutional Neural Network

CNN is a type of artificial neural network that requires a convolutional layer but can have other types of layers, such as nonlinear, pooling, and fully connected layers, to create a deep convolutional neural network [19, 22, 26, 27]. Depending on the application, CNN can be beneficial [20]. However, it brings additional parameters for training. In the CNN, convolutional filters are trained using the backpropagation method. The shapes of the filter structure depend on the given task. For example, in an application such as face detection, one filter can perform edge extraction, whereas another can carry out eye extraction. However, we do not fully control these filters in CNN, and their values are determined through learning [19, 28–30]. This section briefly introduces the CNN.

#### 2.2.1. Convolutional Layer

**Figure 2:**
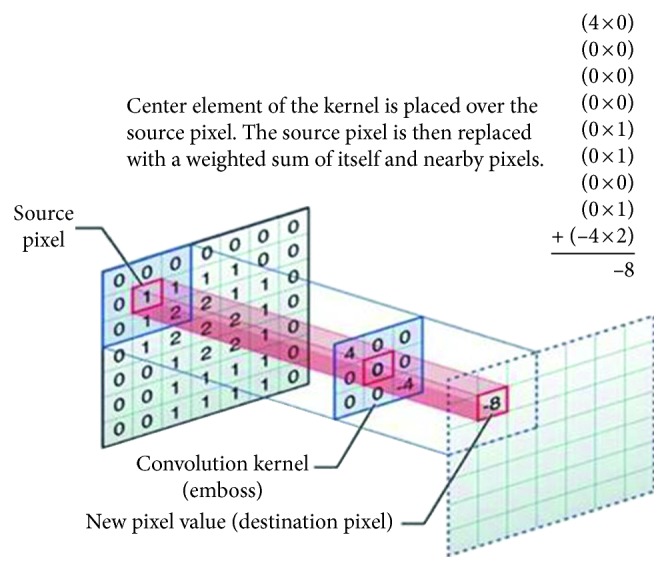
Convolution layer slides the filter over a given input. Output is the summation of an element by the element matrix multiplication of the filter and receptive field (image from [23]).

In the convolutional layer, multiple filters slide over the layer for the given input data. A summation of an element-by-element multiplication of the filters and receptive field of the input is then calculated as the output of this layer. The weighted summation is placed as an element of the next layer.  Figure 2 shows that the filter matrix (middle) is multiplied by the focused area (left matrix), which is denoted by the colors blue and red as its center. Result of this multiplication will be stored in the corresponding place of the center of focus in the next layer. We can then slide the focus area and fill the other elements of the convolution result [22, 27].

Each of the convolutional operation is specified by stride, filter size, and zero padding. Stride, which is a positive integer number, determines the sliding step. For example, stride 1 means that we slide the filter one place to the right each time and then calculate the output. Filter size (receptive field) must be fixed across all filters used in the same convolutional operation. Zero padding adds zero rows and columns to the original input matrix to control the size of the output feature map [23, 27, 31].

Zero padding mainly aims to include the data at the edge of the input matrix. Without zero padding, the convolution output is smaller in size than the input. Therefore, the network size shrinks by having multiple layers of convolutions, which limits the number of convolutional layers in a network. However, zero padding prevents the shrinking of networks and provides unlimited deep layers in our network architecture.

#### 2.2.2. Nonlinearity

The main task of using nonlinearity is to adjust or cut off the generated output. Several nonlinear functions can be utilized in the CNN. However, the rectified linear unit (ReLU) is one of the most common nonlinearities applied in various fields, such as image processing [27, 32]. The ReLU can be represented as

(1) $$\begin{array}{c} \text{ReLU}=\left\{\begin{array}{cc} 0, & \text{if }x<0,\\ x, & \text{if }x\geq 0. \end{array}\right. \end{array}$$

#### 2.2.3. Pooling Layer

The pooling layer roughly reduces the dimension of the inputs. The most popular pooling method, max pooling, represents the maximum value inside the pooling filter (2 × 2) as the output [33, 34]. Other pooling methods, such as averaging and summation, are available. However, the max pooling is a widespread and promising method in the literature because it provides significant results by downsampling input size by 75% [30, 35].

#### 2.2.4. Softmax Layer

Softmax layer is considered an excellent method to demonstrate categorical distribution. The softmax function, which is mostly used in the output layer, is a normalized exponent of the output values [36]. This function is differentiable and represents a certain probability of the output. Moreover, the exponential element increases the maximum value probability [29]. The softmax equation is given as follows:

(2) $$\begin{array}{c} o_{i}=\frac{e^{z_{i}}}{\sum_{i=1}^{M}e^{z_{i}}}, \end{array}$$

where *o*_*i*_ is the softmax output number *i*, *z*_*i*_ is the output *i* before the softmax, and *M* is the total number of output nodes.

## 3. The Proposed Method

Our approach uses CNN and raw sensor data to classify social gestures. The main challenge is finding an optimal architecture for the CNN. Therefore, we first defined the input and output structures of the network. We then presented the optimal architecture based on the results of various experiments. Each recorded sample is an 8 × 8 × *N* matrix. However, the frame length (*N*) is variable for each sample because each subject uniquely carries out social gestures. For an effective implementation, the input size should be fixed. One process assumes the input size according to the sample with the maximum frame length and utilizes zero padding (at the end of recorded samples) for those with short frames. However, this process provides CNN with a huge input size (i.e., 512 × 8 × 8) and is computationally expensive. Another method that we implemented is splitting the samples with a fixed length, which means dividing each sample into subsamples. Whenever the number of frames is not completely divisible by the given subsample length, the reminder of the subsample which has less frame number than the given one is padded by zeros. The results will determine the optimal frame length. This method poses the following advantages:

- The number of samples used to train the neural network increases, which, however, depends on the frame length. Short frame lengths denote additional subsamples with less information, and vice versa.
- We obtained subsamples derived from another part of the main sample (i.e., from the middle or toward the end of social gestures). Thus, our method can recognize the social gesture class although it is unspecified in the beginning.
- The proposed methods in previous studies were not designed for real-time classification. Rather, these methods recognize class after the gesture is completed. By contrast, our approach recognizes the gesture after receiving a fixed length of data.

The softmax function with 14 classes is the output shape of our method. Although we employed the peak value in the output node as the calculated class, we relied on the softmax values to consider other highly probable hypotheses.

Our approach is similar to that used in CNN for video classification or image processing. Image classification uses color images, in which the input shapes are, for example, 128 × 128 × 3. The output shape of our social gesture recognition is 8 × 8 × *N*, where *N* denotes the frame length. In CNN, The number of output feature map from the convolutional stage is equal to the number of used filters. So, increase in the filters which are used at the convolutional layer leads to increase in the output feature maps. Increasing the frame length leads to increase in the input channels, thereby adding convolution operations, which are computationally expensive as previously mentioned.

We cascaded the convolutional layers together to build the classifier. Each convolutional layer consists of convolution, nonlinearity, and pooling. We proposed three convolutional layers with one fully connected layer and, lastly, softmax for our gesture recognition system. The meta parameters of our CNN architecture are presented in Table 1.

## 4. Results and Discussion

**Figure 3:**
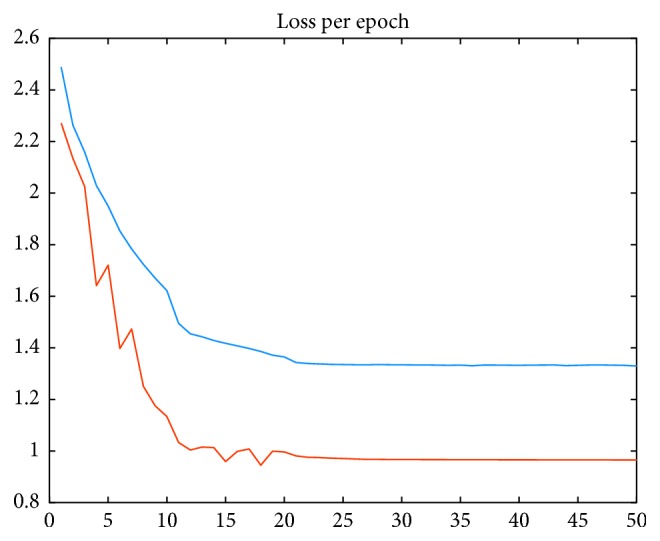
The loss/objective as a function of training epoch.

For touch gesture recognition, we used MATLAB (Release 2016a) and LightNet Toolbox as a versatile and purely Matlab-based environment for the deep-learning framework [37]. The number of epochs is set to 50 and batch size to 250. The simple stochastic gradient descent (SGD) is used as a learning function. However, the momentum term is set to 0.9 and learning rates are selected as 1, 0.5, 0.1, 0.05, and 0.001; in every 10 epochs, we search for a new learning rate over a batch of data and we select the learning which gives the minimum loss.  Figure 3 shows the loss/objective as a function of training epoch; red and blue curves indicate the training and test loss values, respectively. A grid search is performed to select the optimal number of frame length.

**Figure 4:**
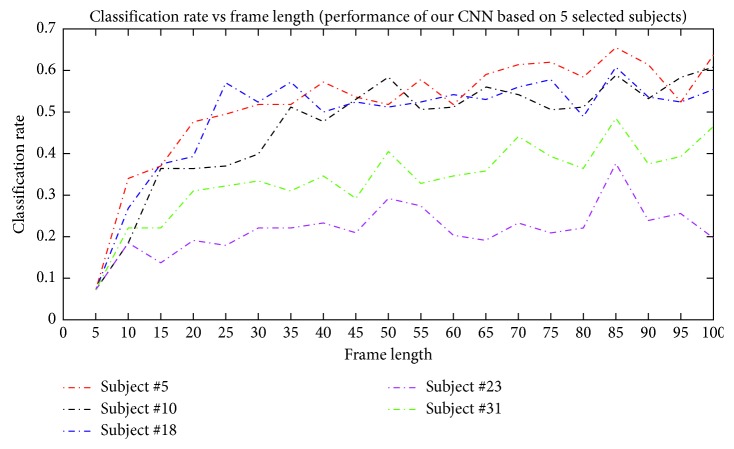
Evaluation of the performance of CNN with three convolutional layers using five randomly selected subjects from the CoST dataset. The figure shows improved performance with the increase in the frame length of the data.

We ran the experiments for frame lengths of 5, 10, 15, 20,…, 100 to compute the optimum frame length. Five random subjects were selected for the hold-out validation test to find the hyperparameters (due to the computationally expensive experiments). These subjects' IDs are 5, 10, 18, 23, and 31. The criterion for the optimal frame length is the average cross-validation accuracy. The results for the subjects and their average are shown in Figure 4. In summary, increasing frame length improves the classification rate. Also, using less than 30 frames, which are equivalent to 222 ms, leads to poor performance. This number seems to add sufficient content to the samples. However, our system performance was not equivalent to 222 ms, leading to poor performance. This number seems to add sufficient content to the samples.

However, our system performance did not importantly increase after 40 frames. The proposed system achieved the maximum classification rate at 85 frames, which are equivalent to 629 ms. Thus, this value is selected as the input dimension of our CNN.

Results of the leave-one-subject-out cross validation for all subjects ranged from 39.1% to 73% (*M* = 63.7%; SD = 11.852%) as presented in Table 2, which outperforms state-of-the-art results.

To further understand the results, Table 3 presents the confusion matrix. The table shows few large nondiagonal numbers, which indicate a major confusion in our proposed method. In addition, mutual confusion exists in the following classes: grab and stroke, massage and stroke, hit and slap, pat and tap, rub and squeeze, rub and scratch, rub and press, and tickle and scratch.

**Figure 5:**
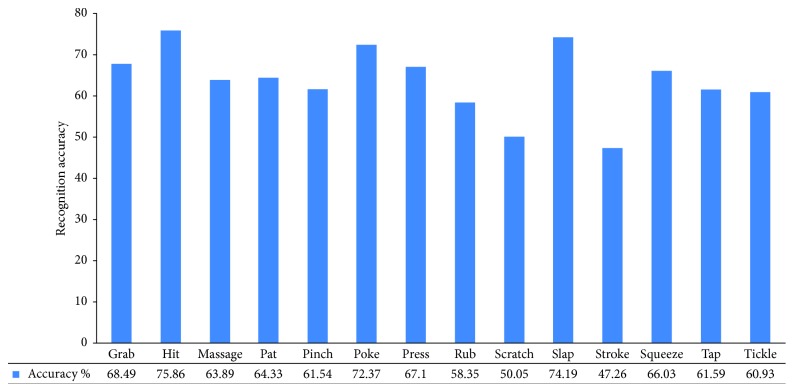
Accuracy of the proposed model in predicting each gesture class.

Indeed, the confusion makes sense because these gestures are similarly performed by humans. An interesting outcome is that important confusion is lacking between massage and grab despite both being confused with stroke. The same is true for tickle and rub, which has a mutual confusion with scratch. The performance of the proposed system is slightly different based on the gesture class as explained in Figure 5. The least accurate classification is for stroke and scratch. These two classes have multiple mutual conflicts with other classes, whereas the peak performance belongs to the hit class. We compare the performance and results of our method with other existing methods for touch recognition gestures.

In comparison with the previous work, we have addressed more challenging tasks which is classifying a gesture using its subsamples. Although we used less number of samples, we did not use the whole length of the sample for prediction. Moreover, in our approach with subsampling, we have generated more data for training and more tests for evaluation. A subsampling of 85 frames (with 10 frames sliding samples) generates about 15 times more test sets which of course is more challenging.

The leave-one-subject-out cross validation is used to evaluate the classification accuracy of CNN for CoST dataset. However, other previous approaches utilized 21 subjects for training and 10 subjects for testing. Thus, training on the additional nine subjects would likely result in improved performance.  Table 4 illustrates the comparison between the proposed and other classification algorithms applied on the CoST dataset. The proposed algorithm improves the correct classification ratio (CCR) without preprocessing, which depends on the original input data instead of feature extraction. However, this process introduces loss of certain information from raw data.

## 5. Conclusion

In this paper, a system that classifies touch gesture in nearly real time using a deep neural network is proposed. The CNN is presented, which is considered a good feature extractor algorithm. The CoST dataset was used to train our CNN for various classes. Results showed that our method performed better compared with the previous work based on leave-one-subject-out cross validation for the CoST dataset.

The proposed approach poses two benefits compared with those in the existing literature. First, the proposed method does not need data preprocessing or manual feature extraction and can be applied end-to-end. Second, this method can recognize a class after receiving a minimum number of frames. This minimum number of frames can be provided by the CoST dataset using grid search. Meanwhile, the proposed approach also has certain limitations. First, CNN performance is affected by the size of the input frame. The smaller the size of the frame (8 × 8 pixels), the more negative the effect on CNN performance because CNN behavior reduces the size of input data in the subsequent layers. Thus, zero padding for rows and columns of a frame is utilized after convolutional operation to repair the lost frame size before pooling operation. Second, increasing the number of filters used in convolutional operation improves CNN performance. However, the time consumed to train the network will be increased.

## Figures and Tables

**Table 1:** Meta parameters of our CNN architecture.

Layer number	Element	Parameter	Value
1	Convolutional filter	Input channels	8 × 8 × 85
		Size	3 × 3
		Stride	1
		Pad	1
	Max pooling	Size	2 × 2
		Pad	2

2	Convolutional filter	Input channels	64
		Size	2 × 2
		Stride	1
		Pad	1
	Max pooling	Size	2 × 2
		Pad	2

3	Convolutional filter	Input channels	128
		Size	3 × 3
		Stride	1
		Pad	1
	Max pooling	Size	2 × 2
		Pad	2

4	Fully connected	Input to layer	256 × 2 × 2
		Output from layer	512

5	Softmax	Output units	14

**Table 2:** The average leave-one-subject-out cross-validation result using our proposed CNN for the gesture recognition.

The validation method	Correct classification rate (CCR)	Standard deviation
Leave-one-subject-out	63.7%	11.852%

**Table 3:** Results of our proposed CNN for gesture recognition presented as the accumulated confusion matrix of the leave-one-subject-out cross validation for all subjects.

Gesture	1	2	3	4	5	6	7	8	9	10	11	12	13	14	Total
Grab (1)	552	2	50	1	12	3	12	4	9	1	146	8	1	7	808
Hit (2)	1	198	5	7	7	7	4	2	5	27	4	1	15	6	289
Massage (3)	35	1	1090	3	37	8	38	140	57	1	103	49	2	102	1666
Pat (4)	5	7	8	220	4	3	6	8	21	14	9	9	37	37	388
Pinch (5)	9	1	42	1	392	25	14	9	15	2	48	16	1	21	596
Poke (6)	7	2	8	3	24	296	21	3	11	1	9	6	11	22	424
Press (7)	32	1	36	2	18	17	461	32	10	2	21	14	5	14	665
Rub (8)	13	0	125	7	15	3	72	580	170	3	15	125	1	79	1208
Scratch (9)	9	2	70	13	11	9	17	73	523	4	8	35	7	188	969
Slap (10)	1	31	4	15	3	3	5	4	8	230	9	8	16	12	349
Stroke (11)	126	1	116	2	57	2	17	10	7	3	345	7	1	21	715
Squeeze (12)	4	1	75	7	23	2	8	94	41	2	4	591	3	76	931
Tap (13)	2	10	8	48	5	11	4	7	13	14	0	7	186	47	362
Tickle (14)	10	4	69	13	29	20	8	28	155	6	9	19	16	986	1372
Total	806	261	1706	342	637	409	687	994	1045	310	730	895	302	1618	10 742
CCR%	68.5	75.86	63.89	64.33	61.54	72.37	67.1	58.35	50.05	74.19	47.26	66.3	61.6	60.93	63.71

**Table 4:** Comparison of features from other existing classification methods applied on same dataset.

No.	Reference	Features extracted	# Subject	# Touch	# Features	Classify method	Accuracy (%)	S.D. (%)
1	[8]	Yes	31	14	28	Bayesian classifier	53	11
						SVM	46	9
2	[9]	Yes	31	14	28	Bayesian classifier	54	12
						SVM	53	11
3	[10]	Yes	31	14	45	Neural network	54	15
4	[11]	Yes	31	14	42	Random forests (RF)	55.6	13
5	[12]	Yes	31	14	5 set	Random forests (RF)	59	
						Boosting	58	
6	[13]	Yes	31	14	7	Deep autoencoders	56	
7	[15]	Yes	31	14	273	SVM	60.5	
						Random forests (RF)	60.8	
8	[17]	Yes	31	14	54	Bayesian classifier	57	11
						Decision tree algorithm	48	10
						SVM	60	11
						Neural network	59	12
9	[24]	No	31	14	Raw data 8×8×45	CNN	42.34	
					Raw data 8×8×45	CNN-RNN	52.86	
					7	Deep autoencoders	33.52	
10	Our proposed method	No	31	14	Input data (raw data) 8×8×85	Convolutional neural network	63.7	11.85

## Data Availability

The data used to support the findings of this study are available from the corresponding author upon request.
